# Perspectives on the clonal persistence of presumed ‘ghost’ genomes in unisexual or allopolyploid taxa arising via hybridization

**DOI:** 10.1038/s41598-019-40865-3

**Published:** 2019-03-18

**Authors:** P. J. Unmack, M. Adams, J. Bylemans, C. M. Hardy, M. P. Hammer, A. Georges

**Affiliations:** 1 Institute for Applied Ecology, University of Canberra, Canberra, ACT 2601 Australia; 2 Department of Biological Sciences, University of Adelaide, Adelaide, SA 5005 Australia; 3 CSIRO Land and Water, GPO Box 1700, Canberra, ACT 2601 Australia; 4 Museum & Art Gallery of the Northern Territory, Darwin, Northern Territory 0810 Australia

## Abstract

Although hybridization between non-sibling species rarely results in viable or fertile offspring, it occasionally produces self-perpetuating or sexually-parasitic lineages in which ancestral genomes are inherited clonally and thus may persist as ‘ghost species’ after ancestor extinction. Ghost species have been detected in animals and plants, for polyploid and diploid organisms, and across clonal, semi-clonal, and even sexual reproductive modes. Here we use a detailed investigation of the evolutionary and taxonomic status of a newly-discovered, putative ghost lineage (HX) in the fish genus *Hypseleotris* to provide perspectives on several important issues not previously explored by other studies on ghost species, but relevant to ongoing discussions about their detection, conservation, and artificial re-creation. Our comprehensive genetic (allozymes, mtDNA) and genomic (SNPs) datasets successfully identified a threatened sexual population of HX in one tiny portion of the extensive distribution displayed by two hemi-clonal HX-containing lineages. We also discuss what confidence should be placed on any assertion that an ancestral species is actually extinct, and how to assess whether any putative sexual ancestor represents a pure remnant, as shown here, or a naturally-occurring resurrection via the crossing of compatible clones or hemi-clones.

## Introduction

Biologists across many fields regard the ‘death’ of a species as a landmark event, and thus species extinction has been the subject of much theoretical and practical research in ecology and evolution^1,2^. Although a few researchers have speculated on how we might resurrect iconic extinct species such as mammoths, thylacines, and dodos^3^, most see extinction as a final, irreversible endpoint. However, there is a category of little-considered exceptions, namely that of ‘ghost’ or ‘semi-living fossil’ species. These are extinct species whose intact genomes are perpetuated via clonal reproduction in unisexual or allopolyploid taxa arising from an historical hybridization event involving that extinct ancestor. This phenomenon is exemplified by a recent report by Dubey and Dufresnes^4^ on the discovery of a ‘ghost’ vertebrate, namely a hybridogenetic lineage of water-frog (*Pelophylax hispanicus*) that clonally transmits the haploid genome of an extinct species (*Pelophylax* n. t. 1) by sexual parasitism of a congeneric sexual species. As discussed by these authors, the potential for de-extinction without genetic engineering is a far more realistic prospect for such ghost species than for fully-extinct taxa.

**Figure 1:**
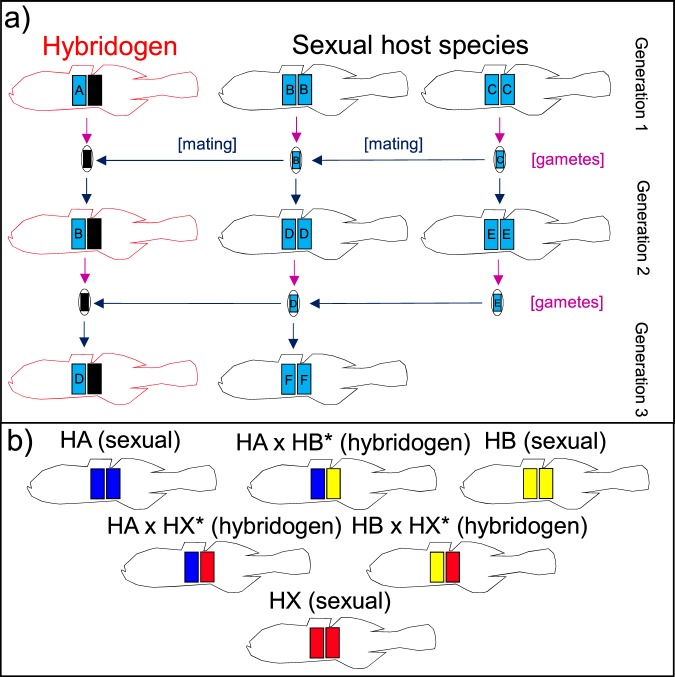
Diagrammatic summaries of hybridogenesis in carp gudgeons. (**a**) Simplified depiction of how haploid genomes (colored rectangles; clonally-transmitted genome in black) are inherited in hybridogenesis versus sexual production for diploid vertebrates. Unique genomes are labelled alphabetically. Note that the sexual host becomes a genetic parent but never a genetic grandparent of its offspring. (**b**) Summary of genomic constitution of each host species and their sexual parasites in *Hypseleotris*. Genome colors match those used in Fig. 2.

While the hemi-clonal nature of hybridogenetic reproduction (Fig. 1a) is perhaps the most striking and bizarre mechanism by which the genomes of extinct species can be perpetuated after their ‘death’, there are several other ways in which such ghost species can persist and therefore be detected in ‘living’ species. Notable among these are the two other categorical modes of ‘unisexual’ reproduction (as defined by Dawley^5^; for an overview of various analogous terms used for plants, see Mirzaghaderi and Hörandl^6^). Gynogenesis and parthenogenesis (plus numerous variants on the three major forms of unisexual reproduction^7^) both have the potential to clonally perpetuate the genomes (both nuclear and/or matrilineal) of extinct species. Reflecting this, evidence for the presence of ‘ghost’ species has also been found or inferred in several other complexes, both for vertebrates^8,9^ and invertebrates^10^.

There is one additional evolutionary pathway by which a now-extinct species can also leave a ‘genomic echo’ of its former existence – the phenomenon of allopolyploid speciation. Here a new polyploid species is ‘instantaneously’ created via hybridization between two related species. Such new species retain both sets of parental chromosomes, are self-perpetuating but reproductively isolated from either parent species, and often pass each ‘original’ parental genome down through successive generations without between-genome recombination^11^. In this latter scenario, ghost species will persist even after the extinction of either parental species for as long as recombination between the parental genomes is suppressed. Allopolyploid speciation is considered rare in animals (although many unisexual lineages are themselves allopolyploid^5^) but is commonplace in plants^12^. Genetic evidence for ghost species has been inferred in allopolyploid frogs^13^, appears implicated for a polyploid parthenogenetic crayfish^14^, and exists for a variety of allopolyploid plants, including those that reproduce asexually via apomixis^15,16^ and sexually^17^.

In this paper we explore in more depth three themes arising from the study of Dubey and Dufresnes^4^. First, we respond to their call “… for the tracking of “ghost” vertebrate lineages that remained hidden and protected in other species of hybrid origins…” by conducting a detailed analysis of two newly-discovered hybridogenetic lineages of Australian freshwater fish in which the clonally-transmitted genome appears to represent a ghost species^18^. Thereafter, we consider two additional questions not addressed by Dubey and Dufresnes^4^. How do we know a ghost species is actually extinct and not just overlooked because it is highly range-restricted, or because of inadequate field or genetic surveys? Finally, given the potential in some organismal groups for the spontaneous resurrection of a ghost species via naturally-occurring hybridization between two ghost-genome-containing lineages, as observed occasionally in waterfrogs^19^ and as speculated for our target organism^18^, how do we know that any presumed-extinct ancestor, once discovered, is actually the ‘pure’ sexual parent species and not a re-birth? Together these considerations ensure our study has obvious implications for research on other apparent ghost species across all eukaryotic groups and regardless of their mode of origin. In particular, such considerations are highly relevant to any de-extinction proposal for a presumed ghost species.

**Figure 2:**
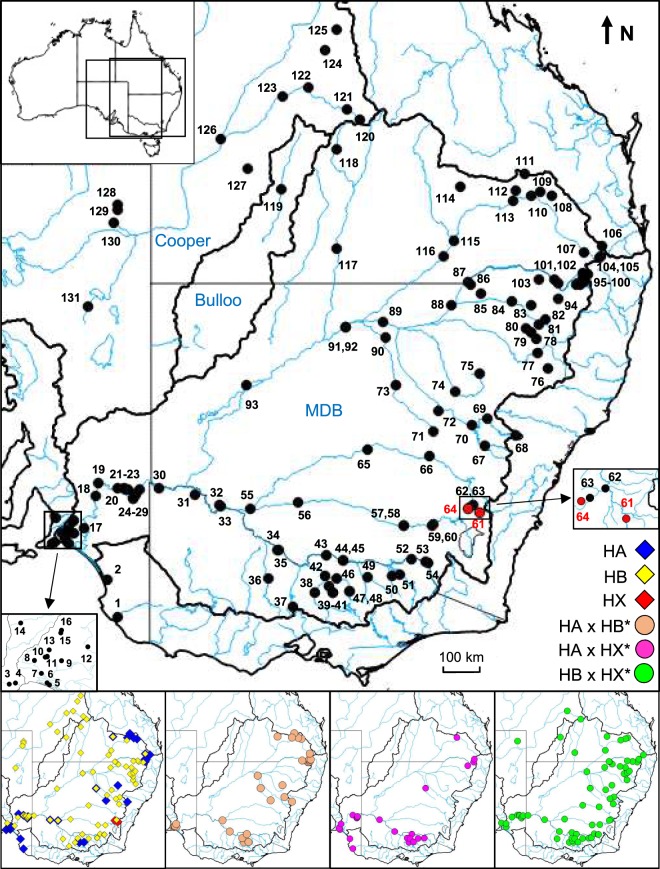
Composite map showing the geographic arrangement of all 131 sites where members of the *Hypseleotris* sexual/unisexual complex were genotyped for this study. Locality details are presented in Table S1. Also shown at the bottom are the comparative distributions for each of the six taxa identified, using the legend supplied.

Our target ‘species’ is a prominent but little-studied fish, the informally-named ‘Lake’s carp gudgeon’ (Eleotridae: *Hypseleotris* sp. 5^20^), which is found throughout much of south-eastern Australia including the Murray-Darling Basin across an area of over 1.4 M km^2^ (Fig. 2). While not formally-described, the taxonomic validity of Lake’s carp gudgeon has long been accepted by the Australian ichthyological community^21^, mostly owing to a very distinctive, easily-identified morphotype not found in any other congeneric species, i.e. the complete absence of scales on the head. It co-occurs throughout its extensive range with three other species of *Hypseleotris*, only one of which has been formally named: western carp gudgeon *H*. *klunzingeri*, Midgley’s carp gudgeon, and the Murray Darling carp gudgeon^21,22^. The genus represents one of the most abundant and widespread groups of native fish in eastern Australia, especially in the vast but highly human-modified Murray-Darling Basin (Fig. 2).

The existence of unisexual lineages in *Hypseleotris* was first proposed by Bertozzi *et al*.^23^. Their allozyme study of fish from five sites across the lower Murray (Fig. 2) revealed the presence of three, often-sympatric sexual species (designated as HA, HB, and HC; reflecting H for *Hypseleotris*, and A, B, C for each species identified) plus every possible F_1_ hybrid combination (the classic genetic signature of vertebrate unisexual lineages^5^) involving HA, HB, and a fourth hypothesised sexual species (HX) that was not found at any site (Fig. 1b). All these findings were confirmed by a subsequent microsatellite and mtDNA study^18^ on 14 populations in an upstream region of the Murray system (Fig. 2), which also provided two additional insights into the group. First, all three unisexual lineages (HA × HB*, HA × HX*, and HB × HX*, Fig. 1b; the asterisk denotes which genome is transmitted clonally) achieved their sexual parasitism through hybridogenesis rather than gynogenesis. Second, while males are rare or absent in most vertebrate unisexual lineages^5,24^, they were the dominant sex in one lineage (98%; HA × HB*) and relatively common in another (24%; HB × HX*).

Both founding genetic studies on the *Hypseleotris* sexual/unisexual species complex demonstrated that the sexual species HX was only detected as a ghost lineage in HA × HX* and HB × HX* hybridogens for sites scattered across more than 1000 river kilometres. This intrigue about whether HX was yet another example of a now-extinct ancestral species, or still persisted as an extant but undiscovered species, was one of the key triggers for ongoing efforts by our research team to undertake combined genetic, ecological, and evolutionary studies on the entire *Hypseleotris* complex. Here we can now report, after an initially-fruitless, decade-plus search, that we have tracked down two extant populations of sexual HX. Importantly, these HX populations have an extremely restricted distribution, amounting to just a tiny fraction of the very broad range occupied by the Lake’s hybridogens (Fig. 2). Thus, while there are critical conservation concerns about the status of this newly-discovered species, reports of its demise are premature and the HX ghost observed in hybridogens is instead more of a ‘genomic shadow’. We also use our genomic dataset to conclude that HX is a pure remnant population of the ancestral parent species, not a resurrected natural cross between two HX-containing hemi-clones.

## Materials and Methods

### Taxonomic and geographic sampling

We intensively and broadly sampled all relevant *Hypseleotris* spp. across the entire documented range of Lake’s carp gudgeon (Murray-Darling Basin, Bulloo River and Cooper Creek catchments; Fig. 2). Sampling was guided by two key observations – (1) initial morphological diagnoses revealed that not all HX-containing hemi-clones consistently displayed the Lake’s carp gudgeon morphotype^23^, and (2) all preliminary and final genetic datasets confirmed previous results that the distinctive sexual species *H*. *klunzingeri* (species HC^23^) is not involved in or a member of this sexual/unisexual complex (data not presented). Our sampling efforts ultimately produced fish from 131 sites, and this extensive collection was the source of the specimens used in our detailed genetic analyses (allozymes and mtDNA). Thereafter, a subset of genotyped fish was used to generate our high-coverage genomic dataset (SNPs). Further insights into the distribution of taxa in and around South Australia were provided by an historic dataset of 860 *Hypseleotris* spp. (including n = 105 from the study by Bertozzi *et al*.^23^) which we had previously collected and allozyme-genotyped between the years 2001–2009. The geographic and taxonomic details of all specimens screened, along with sample sizes employed for each molecular dataset, are contained in Tables S1 and S2. Further details regarding field collection and laboratory handling protocols can be found in Supplementary Information 1. All experimental protocols were approved by the University of Canberra animal ethics committee and the methods were carried out in accordance with the relevant guidelines and regulations.

### Allozyme laboratory procedures and analysis

The primary allozyme dataset comprised the genotypes of 306 fish from 82 sites for 54 putative allozyme loci (including the full suite of 35 loci screened by Bertozzi *et al*.^23^). Fish were chosen to maximise the number of sites surveyed, to include all morphotypic forms at each site, and to increase sample sizes at a few significant sites. We used Principal Co-ordinates Analysis (PCoA^25^) on this dataset to provide an objective diagnostic procedure for identifying lineages from first principles (following the rationale in Adams *et al*.^26^). Fish included in the historic allozyme dataset were identified to taxon using a suite of eight enzymes, shown by Bertozzi *et al*.^23^ and in subsequent screening to be diagnostic for all sexual and unisexual lineages. All enzyme details, laboratory protocols, and analytical procedures for the two allozyme datasets are detailed in Supplementary Information 1.

### SNP methods and analyses

DNA was extracted by Diversity Arrays Technologies (DArT Pty Ltd, Canberra, Australia) and sequenced using DArTseq™ (DArT Pty Ltd) after complexity reduction with restriction enzymes PstI (recognition sequence 5′-CTGCA|G-3′) and SphI (5′-GCATG|C-3′) in a double digest (see Supplemental Materials). Poor quality sequences were first filtered (minimum barcode Phred score 30, pass percentage 75; minimum whole-read Phred score 10, pass percentage 50) to yield approximately 2,000,000 (±7%) sequences per sample, used in marker calling. The error-corrected sequences were analyzed using DArT Pty Ltd proprietary software (DArTsoft14) to output candidate SNP markers. One third of samples were processed twice from DNA to allelic calls as technical replicates, and scoring consistency (reproducibility) was used as the main selection criterion for high quality/low error rate markers.

The SNP data and associated metadata were read into a genlight object ({adegenet}^27^) to facilitate processing with package dartR^28^. Only loci with 99% reproducibility (repAvg) were chosen for subsequent analysis. Further filtering was undertaken by removing any locus with more than 5% missing values (gl.filter.callrate “threshold = 0.95”). Finally, we filtered out secondary SNPs where they occurred in a single sequenced tag, retaining only one SNP at random. Any monomorphic loci arising as a result of the removal of individuals or populations were also deleted. We regard the data remaining after this additional filtering (4,455 SNP markers; 1.51% missing data) as highly reliable. The final filtered dataset is available as Supplementary Data.

Various analyses were undertaken in dartR on this final SNP dataset. As for the allozyme data, the genetic affinities among individuals and populations were visualized using PCoA ordination (gl.pcoa and gl.pcoa.plot functions) to independently delineate all primary taxa from first principles. Levels of observed heterozygosity (H_O_; gl.report.heterozygosity function) were also calculated for all taxa. Finally, for each pairwise comparison of sexual and unisexual taxa, we also calculated the number of SNPs that showed an absolute fixed difference (gl.fixed.diff with tloc = 0) and the Euclidean genetic distance (gl.dist function).

### MtDNA sequencing and analysis

Sequence data for the mitochondrial cytochrome *b* (cyt*b*) gene were also generated to provide information on matrilineal relationships. We included previously published data for 43 individuals from Thacker *et al*.^29^ for Murray-Darling, Bulloo, and Cooper Basin populations (Fig. 2), as well as 13 additional individuals from their sample sites, plus individuals from a selection of the populations sampled for allozyme and/or SNP genotyping. The primary mtDNA dataset comprised 151 fish from 49 sites sequenced for the complete cyt*b* gene, plus 4 *Hypseleotris klunzingeri* as the outgroup. All sequences obtained in this study were deposited in GenBank, accession numbers MK442369–MK442463. Details of our cyt*b* sequencing methods and analyses are presented in Supplementary Information 1.

### Environmental DNA

As part of ongoing monitoring efforts in Blakney Creek, a small tributary of the Lachlan River and located nearby to Meadow Creek (i.e. the site where pure HX was discovered), the fish biodiversity within this system was assessed through environmental DNA (eDNA) metabarcoding. Samples for eDNA analyses were collected during spring 2015 and 2016. Water samples were collected over 19 sampling sites (spanning our sites 62–64; Fig. 2), with eight two litre samples collected per site. Environmental DNA was captured and extracted following the protocols described in Bylemans *et al*.^30^. The universal MiFish-U primers^31^ were subsequently used for eDNA metabarcoding analyses following the general protocols described in Bylemans *et al*.^32^. Full details are in Supplementary Information 1.

## Results

### Taxon identification using allozyme datasets

Although no longer considered cutting-edge, allozymes markers have played a prominent role in the discovery of unisexual taxa^33^ and remain ideally suited for unequivocal taxon and lineage identification^34^, the key starting point for exploring any complex of sexual and unisexual taxa for which reliable morphological diagnoses are currently lacking (i.e. *Hypseleotris* spp, hereinafter defined as excluding *H*. *klunzingeri*). This is because, regardless of their mode of unisexual reproduction, the indicative genetic signature for all unisexual taxa arising via between-species hybridization is the presence of fixed or mostly-fixed heterozygosity at the loci which diagnose the parental species^5^. Moreover, our primary allozyme dataset (306 individuals from 82 sites screened for 54 putative loci) provides a useful baseline comparison for cross-referencing with our other two independent datasets (mtDNA and SNPs).

**Figure 3:**
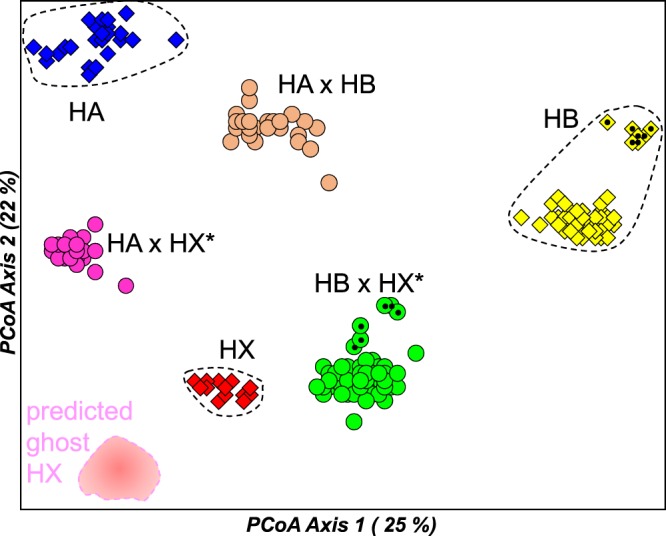
Scatterplot of the relative scores in first two dimensions for the Principal Co-ordinates Analysis of 306 *Hypseleotris*, based on the primary allozyme dataset. Individuals are labelled by taxon with the same symbols as used in Fig. 2. Individuals from the Bulloo and Cooper catchments are additionally identified by a small black dot.

The PCoA for this primary allozyme dataset (Fig. 3), in combination with an examination of the raw allozyme profiles (Table S3), revealed the presence of six major genetic clusters. Of these, five correspond to the two previously-documented sexual species (HA and HB; characterized by low levels of observed heterozygosity and 13 pairwise fixed differences; Tables S3 and S4) plus the three known unisexual lineages (HA × HB, HA × HX*, and HB × HX*; characterized by high levels of observed heterozygosity and largely fixed heterozygosity for the loci diagnostic for their parental species; Table S3). These same five expected genetic groups were also the only ones present for the historic allozyme dataset (860 fish from 23 sites; summary in Table S2).

### Laying a ghost to rest

The remaining sixth cluster comprised 18 fish from a single site (site 61, Meadow Creek) in a small tributary of the upper Lachlan River (Fig. 2). Multiple lines of evidence indicate that this sixth cluster represents a single extant population of the missing sexual species HX. First, its comparative allozyme profile (Table S3) closely matched that predicted by Bertozzi *et al*.^23^ for all key ‘ghost’ allozymes (including being fixed for unique alleles at the three key loci *Fdp*, *Got-1*, and *Me*). Second, this cluster displayed the two aforementioned hallmarks of being a sexual species (low levels of observed heterozygosity and multiple pairwise fixed differences with HA and HB; Tables S3 and S4). Third, both male and female HX were collected, and all displayed the Lake’s carp gudgeon morphotype. Finally, Meadow Creek is the only site where Lake’s carp gudgeon does not co-occur with other taxa within the *Hypseleotris* sexual/unisexual complex, inferring that this population breeds sexually. Thus, we were confident that, 18 years after its discovery as a putative ghost species, we had finally tracked down a remnant population of the HX ancestral species, albeit at only one of more than 130 sites surveyed (Table S2).

One of the advantages of PCoA is that F_1_ hybrids (and therefore *Hypseleotris* unisexuals) should occupy positions on the scattergram that are ‘half-way’ between their parental taxa. While this proved to be the case for HA × HB unisexuals (Fig. 3), the HX-containing unisexuals were somewhat displaced from this half-way position when compared to the extant HX cluster. However, they did occupy the predicted intermediacy when compared to a hypothetical ‘ghost’ HX cluster (Fig. 3), which is qualitative but not definitive evidence that the extant HX population shows at least modest genetic differences from the ancestral HX population(s) originally founding the HX-containing unisexuals. This is consistent with the observation that the widespread sexual species HB itself displayed moderate genetic heterogeneity (4 fixed differences; Nei D = 0.13; Table S4) between sites in the Murray-Darling Basin versus sites from the two inland drainages (Bulloo River and Cooper Creek, Fig. 2), a fact mirrored in the PCoA plots for both inland HB and inland HB × HX*.

### Genomic assessment of the status of HX

The final genomic dataset after filtering comprised 337 fish genotyped for 4,455 SNPs (Table S1). Although the overall percentage of missing values (mostly reflecting mutations at recognition sites for one or both restriction enzymes) was low (1.54%), it was considerably higher for the pure HX population (31.01%). This outcome was repeatable and could not be explained by poor quality DNA, low sequence coverage, or run order. Importantly, all individuals that were genotyped for both allozymes and SNPs were assigned to the same taxon by each molecular dataset (Table S2).

**Figure 4:**
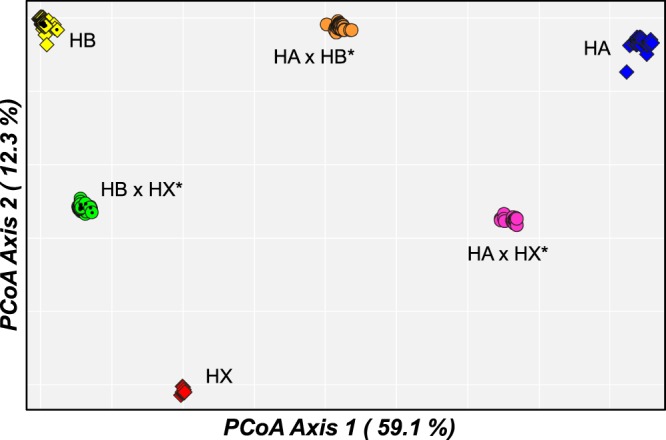
Scatterplot of the relative scores in first two dimensions for the Principal Co-ordinates Analysis of 337 *Hypseleotris*, based on the filtered SNP dataset. Symbols used match those for Fig. 2.

Ordination of the genomic data (Fig. 4) produced a very similar PCoA plot to that obtained for the allozyme data (Fig. 3). Again, there is some indication that the two HX-hybridogens do not occupy PCoA positions that are strictly intermediate between the pure HX population and the relevant parental taxon (in contrast the placement of HA × HB*, which is exactly intermediate), further suggesting the ancestral HX population involved in founding these hybridogens is genetically distinctive from extant HX in Meadow Creek.

Both nuclear datasets have two additional features in common. First, they demonstrate the presence of a major phylogeographic break within HB between MDB sites and those from the two inland drainages (Bulloo River and Cooper Creek; Fig. 2). This dichotomy is not evident in the first two dimensions of the genomic PCoA but is clearly displayed in deeper dimensions (not shown) and confirmed by the presence of absolute fixed differences at 34 SNP loci (Table 1). Second, as for the allozyme data (Table S3), the SNP data demonstrate that, as expected, all unisexual lineages display highly elevated values of H_O_ (range 0.171–0.369; Table 1) when compared to their sexual relatives (range 0.012–0.036).

**Table 1:** Summary of levels of observed heterozygosity and pairwise genetic differences among taxa for the genomic dataset.

Taxon	Observed Heterozygosity	HA	HB MDB	HB inland	HX	HA × HB*	HA × HX*	HB × HX* MDB	HB × HX* inland
		(39)	(119)	(32)	(7)	(37)	(18)	(70)	(15)
HA	0.023	—	40.13	41.99	39.88	20.96	17.14	38.78	39.97
HB (MDB)	0.036	747	—	17.16	30.25	19.92	34.40	12.57	18.41
HB (inland)	0.012	1063	34	—	32.65	24.88	36.59	20.17	13.64
HX	0.014	724	295	611	—	29.98	23.36	17.49	19.38
HA × HB*	0369	0	0	85	325	—	18.98	20.61	23.74
HA × HX*	0.260	0	457	756	91	0	—	29.89	31.42
HB × HX* MDB	0.171	683	0	76	27	0	387	—	12.92
HB × HX* inland	0.176	856	18	0	58	38	519	35	—

Lower matrix = number of absolute fixed differences (total number of SNPs = 3,383 for all comparisons involving HX, and 4,455 for all other comparisons). Upper matrix = Euclidean genetic distances.

### Matrilineal genealogy for the *Hypseleotris* complex

**Figure 5:**
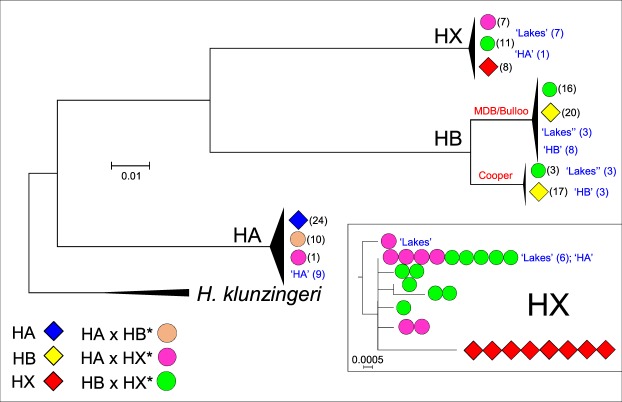
Maximum Likelihood summary tree, rooted using the outgroup *H*. *klunzingeri*. Fish genotyped to actual taxon using either nuclear dataset bear the same symbols as used in Fig. 2, while fish identified solely on morphological criteria are denoted in blue font (although all HB × HX* and many HA × HX* fish display the ‘Lakes’ phenotype, some HA × HX* fish are phenotypically similar to HA). The actual haplotype tree for the HX clade is shown in the inset.

The cyt*b* dataset consisted of 1141 bp for 151 individual ingroup fish (Table S1). Some of these (32 fish, mostly from Thacker *et al*.^29^) lacked corresponding nuclear genetic identifications and thus were only identified by their morphological phenotype. For the ingroup sequences 933 of the 1141 bp were constant, 15 variable characters were parsimony uninformative, and 193 characters were parsimony informative. ML recovered one tree with an ln score of −3311.971219 (full tree in Fig. S1), a summary of which is presented in Fig. 5.

The matrilineal phylogram confirms the *Hypseleotri*s sexual/unisexual complex encompasses three primary matrilineal clades, corresponding to the sexual species HA, HB and HX. In addition to these primary clades, there was an obvious phylogeographic dichotomy within HB, although the split observed (MDB/Bulloo River versus Cooper Creek) was not fully concordant with that identified by both the allozyme and genomic data (MDB versus Bulloo/Cooper). All but one of 90 fish identified as ‘sexuals’ (69 genotyped, 21 identified morphologically) could be assigned to the expected mtDNA clade. Only a single fish with the HA morphotype failed to be assigned to the expected mtDNA clade, instead being in the HX clade. This outcome reflects the fact that some HA × HX* individuals display a phenotype similar to HA when identified in the field (and is consistent with the taxon expectations at this site (#32, see Table S2). None of the 61 sexually-parasitic hybridogens (48 genotyped, 21 identified morphologically) were assigned to a mtDNA clade that was inconsistent with either parental ancestry or known host sexual species.

Two results in the mtDNA analysis are of special significance with respect to the status of the remnant HX population. First, the single haplotype displayed by all 8 HX fish from Meadow Creek was not displayed by any HX-containing hybridogen. Second, this haplotype was clearly the most distinctive of the 8 haplotypes detected in the ‘HX’ clade.

### Pure remnant population or natural de-extinction event?

While all our genetic and genomic analyses confirm the discovery of a population of the sexual species HX in Meadow Creek, we have yet to rule out the possibility that this population has been resurrected through a natural cross between two HX-hybridogens (e.g. male HB × HX* with female HB × HX* being perhaps the most likely cross, given the apparent lack of HA × HX* hybridogens in the Lachlan system; Fig. 2). The key genetic data required to rule this possibility out would be a demonstration that Meadow Creek HX are fixed for alleles or haplotypes that are absent from HX-hybridogens. An analysis of the pairwise genetic distance matrix among all taxa for the SNPs (Table 1) confirms this to be true. These data demonstrate that while there are no absolute fixed differences between any of the three hybridogenetic lineages and the relevant sexual relative for taxa HA and HB (e.g. HA × HB* versus HA and HB; HA × HX* versus HA; HB × HX* versus HB for both lineages of HB), there are numerous fixed differences between Meadow Creek HX and all HX-hybridogens (range 27–91; Table 1). This unequivocally demonstrates that the Meadow Creek site house a natural remnant population of HX. This finding is further supported by the mtDNA results, which indicated that the Meadow Creek HX have a unique and distinctive ‘HX’ cyt*b* haplotype (Fig. 5).

### Urumwalla Creek HX

Our eDNA survey revealed evidence of a second remnant subpopulation of HX in the upper reaches of nearby Urumwalla Creek, a tributary of Blakney Creek. While downstream sites displayed the eDNA signature of the HB mtDNA clade (indicating the presence of HB and/or HB × HX* fish), water profiles from one upstream region (mapping to site 64, Fig. 2; sampling sites UC03–UC05; Fig. S2) only displayed the eDNA profile for the HX mtDNA clade (potentially indicating the presence of HX, HA × HX*, or HB × HX*). Given that both hybridogenetic lineages can only persist via the sexual parasitism of one of their sexual congeners, the absence of HA and HB mtDNA haplotypes indicates that these sites either harbour pure HX sexuals or a mix of HX and one or both hybridogens.

## Discussion

Our comprehensive genetic, genomic and geographic survey of Lake’s carp gudgeon and its sexual and unisexual relatives has delivered a number of important outcomes. Foremost among these is the successful discovery, at just 2 of 131 sites surveyed, of an extant population of the missing sexual ancestor HX. Previously, Lake’s carp gudgeon had always been found to co-occur with other members of this complex^18,22,23^, leading to the reasonable expectation that this ‘species’ only comprised the two known sexually-parasitic hybridogens, both clonally perpetuating the ghost HX genome. Moreover, our genomic and matrilineal datasets clearly demonstrate that this population of HX is definitely a pure remnant of the original sexual species and not naturally-rederived by a genetic cross between existing hemi-clones. The pure remnant species is an important conservation priority and specific action should be made to prevent extinction of pure HX given it is range restricted and isolated, and any environmental change including drought, pollution and fish introductions could be major threats. Other important outcomes include a robust systematic framework for the entire *Hypseleotris* species complex in this region, and the establishment of a series of genotyped morphological voucher specimens to aid future taxon diagnoses in this abundant keystone group of freshwater fishes, which features prominently in the ecosystem biology of eastern Australia’s waterways^35^.

Our discovery of species HX is an exciting breakthrough for the Australian and world ichthyological community. Not only does it add an additional species to the depauperate and vulnerable freshwater fish fauna of the Murray-Darling Basin, Australia’s most intensively-regulated and economically important river system^36^, it greatly enhances our ability to intensively research the evolutionary origins of unisexuality in *Hypseleotris*. In particular, the availability of all three sexual ancestors provides a huge opportunity to undertake laboratory crosses for all possible within and between-taxon combinations. In conjunction with SNP genotyping of parents and offspring, such insights will allow us to determine which species readily hybridize and if such hybrids are fertile, whether male and female unisexuals can interbreed, if hybridogenesis is the only modal form of sexual parasitism (some sexual/unisexual fish complexes feature both gynogenetic and hybridogenetic lineages^5^), and whether it is indeed possible to artificially recreate sexual fish by crossing the appropriate taxa (e.g., male HA × HB* crossed with female HB; female HA × HX crossed with male HB × HX* etc.).

At the outset we raised the issue of what confidence should be placed on any assertion that a clonally-maintained genome reflects a genuine ghost species i.e. whether the ancestor is actually extinct or simply remains undiscovered or undetected. While we have been successful here in locating the missing sexual ancestor for Lake’s carp gudgeon, any reduced intensity devoted to geographic sampling would likely have failed to collect at the Meadow Creek site (only one of four sites in the upper Lachlan system to harbour HX in our two nuclear datasets). This perspective highlights one of the major caveats that usually applies to the presumption that any taxon has become extinct. Unlike the dodo and passenger pigeon for example, the majority of presumed-extinct species (including HX herein) are characterized by one or more life history attributes (e.g. small size, belonging to a little-studied group, rarity, cryptic behaviour, unobservable unless surveyed using specialized techniques, an inhabitant of hard-to-access habitats^37^) that together cast doubt on any pronouncement of extinction^38^. This point is strikingly illustrated by the discovery in 1938 of a living coelacanth, a ‘fossil species’ previously thought to have become extinct in the Mesozoic era^39^. Stories concerning such high profile ‘Lazarus’ species^40^ are currently popular in the media (e.g. Brown^41^) but are just the tip of the iceberg tip when compared to the large number of low-profile, presumed extinctions that have been proclaimed or speculated upon since Darwin’s time. Given the expected increase in extinction rates predicted into the future^42^, the ambiguity and debates surrounding extinction and re-discovery can only intensify^38,40,43^.

As ‘semi-living fossils’ (*sensu* Dubey and Dufresnes^4^), the same issues apply to ghost species as for any ‘fossil species’ or other presumed-extinctions, with added complications. First, since ghost species are identified on genetic or genomic criteria, researchers cannot know with certainty what the extant but uncollected ancestor actually looks like, and instead must make assumptions based on differences between the ‘ghost-hosting’ unisexual or allopolyploid taxon and the non-extinct parent species. Fortunately, these assumptions proved accurate in this study, since pure HX do indeed show a complete lack of scales from the head to the mid-body region. However, even armed with this knowledge, it is still currently impossible to unequivocally identify pure HX from its two HX-containing hemi-clones (particularly HB × HX*) in the field. Thus, until and unless diagnostic differences can be identified between HX and its hemiclones in a thorough morphological assessment, any future survey to find additional distributional pockets of pure HX will of necessity involve molecular work. Non-invasive genetic monitoring techniques, such as eDNA, can be of particular value in this regard as they can be implemented on a large scale with relative low effort and the method can be optimized to specifically target the genetic signature of ghost species and their congeners. While such an approach can prioritize sampling sites and/or catchments, in-depth whole genome studies would still be needed to unequivocally disprove extinction. This limitation is mirrored in other groups harbouring presumed ghost species^4,16^.

At first glance it may seem odd that only one of the three sexual *Hypseleotris* species is highly range-restricted while the other two are abundant and widespread (Fig. 2). One likely contributing factor here is the very nature of all sexual/unisexual/allopolyploid complexes, particularly those involving sexual parasitism. While most unisexual lineages do not persist over long periods of evolutionary time^7^, they will nevertheless often outcompete a co-occurring sexual relative and may even drive a sexual species to local extinction^44^. Once highly fragmented, that sexual ancestor species will likely find it difficult to maintain gene flow among fragments, particularly where sexual parasitism of multiple sexual species is involved (i.e., in carp gudgeons). In such cases, any between-deme migrants will have to run the gauntlet of unisexual forms that use other sexual congeners to self-perpetuate while effectively acting as ‘biotic barriers’ that lessen the prospects these migrants will successfully mate with other conspecifics.

Any comprehensive demonstration that a presumed pure sexual ancestor has not gone extinct should also be able to rule in or out any ‘resurrection’ scenario, since this knowledge has implications for both conservation and evolutionary perspectives attending any ‘ghost busting’ study. Intriguingly, Schmidt *et al*.^18^ did not find the HB sexual species at any of their study sites, which led them to speculate that the HB × HX* lineage may have been generated by matings between HA × HB* (mostly males) and HA × HX* (mostly females) hybridogens. While two other explanations (HB were present in the region but not sampled due to methodological or habitat-related collection biases; the HB × HX* lineage can also reproduce gynogenetically in some instances) remain tenable, the possibility that crosses between unisexual lineages might produce fertile offspring ensures that the creation of ‘pure’ HX offspring from a cross between HA × HX* and HB × HX* cannot be discounted. Here we have used the power of genomic data, specifically thousands of co-dominant SNPs, to demonstrate that Meadow Creek HX share no alleles with either HX-containing hemi-clone at multiple, unlinked loci (Table 1). This outcome is not consistent with any resurrection hypothesis, since any HX created in this manner must, by definition, share alleles with their founding parental lineages at all loci sampled. We suggest that this ‘genomic fixed differences’ approach offers the most promising way to explore resurrection hypotheses in all relevant sexual/unisexual/allopolyploid complexes.

Given ghost and other ‘living dead’ memes are popular across many human cultures and endeavors, it is not surprising that biologists too are fascinated by the idea that the genomes of extinct species can live on as ghosts, clonally-maintained by their unsexual or allopolyploid relatives. Our study demonstrates the inherent “absence of evidence” dilemma faced when postulating the extinction of any species^38^, and highlights some of the unique issues to consider when that extinct species is also presumed to survive as a ghost. As shown here, some of these issues can be resolved using high-resolution genomics or aided by new technologies such as eDNA, but there remains no technological substitute for the intensive, ongoing field surveys required to lay a ghost to rest.

## Supplementary information

Supplementary Information

Supplementary Dataset 1

## Data Availability

Allozyme and SNP data are provided in the supplementary material, mitochondrial DNA sequences are deposited on GenBank.
