# Supplementary material for: Perspectives on the clonal persistence of presumed ‘ghost’ genomes in unisexual or allopolyploid taxa arising via hybridization
Source: Sci Rep. 2019 Mar 18;9:4730. doi: 10.1038/s41598-019-40865-3 (PMC6426837; doi:10.1038/s41598-019-40865-3)
Supplement: Supplementary file 1 — Supplementary Information [file 41598_2019_40865_MOESM1_ESM.pdf]

Perspectives on the clonal persistence of presumed ‘ghost’ genomes in unisexual or allopolyploid taxa arising via hybridization

Unmack, P.J.*, Adams, M., Bylemans, J., Hardy, C.M., Hammer, M.P., and Georges, A.

*Correspondence to peter.unmack@canberra.edu.au

Supplementary Information, Materials and Methods

Field collection and laboratory handling protocols. All fish were sampled under valid collecting permits issued by the relevant State authorities, and the research was conducted in accordance with animal ethics regulations. Fishes were euthanased with clove oil, then snap frozen in liquid nitrogen and subsequently stored at -70°C at the South Australian Museum. Individuals from the same localities were usually retained as voucher specimens, and have been deposited at the Australian, South Australian and Victorian museums. These samples can be identified based on their station code (Table S1). All genetic analyses employed muscle, subsampled from the caudal peduncle region.

Allozyme laboratory procedures and analysis. Our laboratory protocols are presented in detail by Richardson et al. (1986). The following enzymes or non-enzymatic proteins (EC = Enzyme Commission identifiers) displayed interpretable allozyme patterns when generating the primary allozyme dataset (308 fish; 54 putative loci):- aconitase hydratase (ACON, EC 4.2.1.3), acid phosphatase (ACP, EC 3.1.3.2), adenosine deaminase (ADA, EC 3.5.4.4), alcohol dehydrogenase (ADH, EC 1.1.1.1), adenylate kinase (AK, EC 2.7.4.3), fructose-bisphosphate aldolase (ALD, EC 4.1.2.13), aldehyde dehydrogenase (ALDH, EC 1.2.1.5), carbonate dehydratase (CA, EC 4.2.1.1), creatine kinase (CK, EC 2.7.3.2), enolase (ENOL, EC 4.2.1.11), esterase (EST, EC 3.1.1.), fructose-bisphosphatase (FDP, EC 3.1.3.11), fumarate hydratase (FUM, EC 4.2.1.2), glucose-6-phosphate dehydrogenase (G6PD, EC 1.1.1.49), glyceraldehyde-3-phosphate dehydrogenase (GAPD, EC 1.2.1.12), lactoylglutathione lyase (GLO, EC 4.4.1.5), aspartate aminotransferase (GOT, EC 2.6.1.1), general protein (GP), glycerol-3-phosphate dehydrogenase (GPD, EC 1.1.1.8), glucose-6-phosphate isomerase (GPI, EC 5.3.1.9), glutathione reductase (GSR, EC 1.6.4.2), isocitrate dehydrogenase (IDH, EC 1.1.1.42), cytosol aminopeptidase (LAP, EC 3.4.11.1), L-lactate dehydrogenase (LDH, EC 1.1.1.27), malate dehydrogenase (MDH, EC 1.1.1.37), 'malic' enzyme (ME, EC 1.1.1.40), mannose-6-phosphate isomerase (MPI, EC 5.3.1.8), nucleoside-diphosphate kinase (NDPK, EC 2.7.4.6), dipeptidase [VAL-LEU] (PEPA, EC 3.4.13.), tripeptide aminopeptidase (PEPB, EC 3.4.11.), dipeptidase [LYS-LEU] (PEP-C, EC 3.4.13.), proline dipeptidase (PEPD, EC 3.4.13.), phosphoglycerate mutase (PGAM, EC 5.4.2.1), phosphogluconate dehydrogenase (6PGD, EC 1.1.1.44), phosphoglycerate kinase (PGK, EC 2.7.2.3), phosphoglucomutase (PGM, EC 5.4.2.2), pyruvate kinase (PK, EC 2.7.1.40), L-iditol dehydrogenase (SORDH, EC 1.1.1.14), and triose-phosphate isomerase (TPI, EC 5.3.1.1). Allozymes were designated alphabetically and multiple loci, where present, were designated numerically, both in order of increasing electrophoretic mobility. The following suite of enzymes was used to genotype fish to taxon for the historic dataset: ADA, FDP, FUM, GOT, GPI, ME, PEPD, and PGM. Our methods for undertaking Principal Co-ordinates Analysis follow Adams et al. (2014).

MtDNA sequencing and analysis. The complete mitochondrial cytochrome *b* (cyt*b*) gene was sequenced following the PCR protocols in Hammer et al. (2014), except that samples were amplified with the following primer pairs: Glu18 TAACCAGGACTAATGRCTTGAA with Hd.alt GGRTTGTTGGAGCCTGTTTCAT or Hd.Hyps GGGTTGTTGGAGCCSGTTTCGT and midg.496 GGCGGCTTTTCRGTAGATAA with Eleo.Thr.40 GATTTTAACCTCCTGCGTCCG. Sequences were edited using Chromas 2.6.5 (Technelysium, Tewantin, Queensland, Australia) and imported into BioEdit 7.2.5 (Hall 1999). Sequences were aligned by eye and checked via amino acid coding in MEGA 7.0.18 (Kumar et al. 2016) to test for unexpected frame shift errors or stop codons. The best model of sequence evolution and partition scheme was determined using PartitionFinder 2.1.1

(Lanfear et al. 2016) using PhyML 3.0 (Guindon et al. 2010) based on the following settings: branchlengths = linked; models of evolution = all; model_selection =AICc; partitioned by codon position; search scheme = all. PartitionFinder identified a three partition scheme for our ML analyses by codon position: 1, K80+I; 2, HKY+I and 3. GTR+G. We used GARLI 2.01 (Zwickl 2006) on the CIPRES cluster (Miller, Pfeiffer & Schwartz 2010) to obtain the best Maximum Likelihood tree. The ML analysis was run with 10 search replicates with the following default setting values changed: attachmentspertaxon = 310; genthreshfortopoterm = 100,000; significanttopochange = 0.00001. Trees were rooted using western carp gudgeon (*H. klunzingeri*).

eDNA metabarcoding. Environmental DNA sampling was conducted at 19 sampling sites within Blakney Creek (BC) and its tributary Urumwalla Creek (UC) (Figure S2). Samples were collected over two sampling seasons (i.e. spring 2015 and spring 2016) with eight 2 litre water samples collected at each site. Downstream analyses were performed using all eight eDNA samples per site except for three occasions where, due to loss of a sample or depletion of eDNA extracts, only seven samples were available. To avoid potential contamination all sampling equipment was treated with a 20% bleach solution and thoroughly rinsing with UV-sterilized tap water. Appropriate negative controls were also included to monitor potential contamination and consisted of one blank field control for each site (i.e. a 2 litre sampling bottle filled with UV-sterilized water, opened on site, closed and submerged in the water). Samples were stored on ice and transported back to the University of Canberra (ACT, Australia) where eDNA was captured using a 1.2 µm glass fibre filter (Satorius, Göttingen, Germany). Filtering equipment was cleaned as described previously and a negative equipment controls were obtained for each sample by filtering 500 mL of UV-sterilized water prior to processing the eDNA samples. Filters were stored at -20°C and transferred to the trace DNA laboratory at the University of Canberra for the extraction of eDNA (i.e. following the PowerWater DNA Extraction Kit protocol) and the subsequent processing of samples.

The amplification and the construction of the HTS libraries was done using the MiFish-U universal fish primers Miya et al. (2015). These primers were chosen based on the results of an in-silico evaluation of potential metabarcoding primers for the fish biodiversity present in the Murray-Darling Basin (Bylemans et al. 2018). Negative control samples were first screened for the presence of fish DNA by performing three PCR replicates per sample using the MiFish-U primers and previously described reaction conditions (Bylemans et al 2018). Libraries for High-Throughput sequencing were subsequently prepared for all eDNA samples and the negative controls that tested positive for fish DNA. The construction of libraries for unidirectional sequencing was done using a one-step Real-Time PCR amplifications with fusion tagged primers (FTP). Forward FTP consisted of the P5 sequencing adaptor, a custom forward sequencing primer, a 7 bp Multiplex Identification (MID-) tag and the MiFish-U forward primer. Reverse FTP contained the P7 sequencing adaptor, a 7 bp MID-tag and the MiFish-U reverse primer. MID-tags were generated using the EDITTAG scripts and unique combinations of forward and reverse MID-tags were used to label amplicons from each sampling site (Faircloth and Glenn 2012). Three replicate PCRs were performed per sample and the average Ct-value for each sample was used to pool amplicon libraries from 8 to 10 samples prior to conducting a library clean-up using Agencourt AMPure XP Beads (Beckman Coulter, Brea, USA) in a 1.2 volume ratio. Gel-electrophoresis (i.e. 2% agarose gel with a run

time of 30 min at 120 V) was used to confirm the presence of a single amplicon and amplicon pools were subsequently pooled into a super pool based on the observed band intensities. Finally, amplicon libraries were sequenced using the MiSeq v2 1x300bp sequencing kit at the Ramaciotti Centre for Genomics (University of New South Wales) to achieve an expected read depth of 50,000-60,000 reads per sample.

Processing of the raw sequencing reads followed the workflow described in Bylemans et al. (2018) with some modifications. First, technical sequences (i.e. sequencing adaptors and sequencing primers) were removed using Trimmomatic 0.36 while simultaneously removing low quality bases (i.e. trimming of bases at the end of reads with a quality below 3 and trimming of reads with a sliding window of 4 bases with an average quality threshold of 15) (Bolger et al. 2014). Subsequent filtering of the sequencing reads used the OBITOOLS software package (Boyer et al. 2016). Firstly, the sequencing reads were assigned to their respective sampling site and further filtering was done for each site independently. Short (< 150 bp) and low abundant reads (< 100) were removed before discarding reads arising from PCR and sequencing errors. Taxonomic information was assigned to the sequences using the taxonomic database containing vertebrate sequences from the EMBL data repository (release 132) and 12S sequence from all fishes of the MDB (Bylemans et al. 2018). After the bio-informatics filtering process all fish sequences present in the negative controls, likely to arise from spill-over or low levels of contamination, were removed.

An additional quality check was performed using R 3.4.1 and the tidyverse package (R Development Core Team 2010; Wickham 2017). Some of the sequences were assigned to common consumption fish which are not present within the system (i.e. *Scomberomorus* sp., *Gadus* sp., *Pollachius virens*, *Seriola lalandi* and *Salmo salar*). These occurrences are likely to arise from secondary contamination of the samples and were thus discarded. For one sampling site sequence records were assigned to the genus *Hypseleotris* (ca. 0.14 % of all fish sequences) and *Hypseleotris* sp. 'Murray-Darling carp gudgeon' (ca. 0.04 % of all fish sequences). A closer inspecting of these sequences revealed that they are likely to be chimeric sequences and were thus removed (i.e. combination of *H.* sp. 'Midgley's carp gudgeon' and *H.* sp. 'Lake's carp gudgeon' sequences).

References

Adams, M., Raadik, T.A., Burridge, C.P., Georges, A., 2014. Global biodiversity assessment and hyper-cryptic species complexes: more than one species of elephant in the room? Systematic Biology 63, 518–533.

Bolger, A.M., Lohse, M. and Usadel, B., 2014. Trimmomatic: a flexible trimmer for Illumina sequence data. Bioinformatics 30, 2114–2120.

Boyer, F., Mercier, C., Bonin, A., Le Bras, Y., Taberlet, P. and Coissac, E., 2016. Obitools: a unix-inspired software package for DNA metabarcoding. Molecular Ecology Resources 16, 176–182.

Bylemans, J., Gleeson, D.M., Hardy, C.M. and Furlan, E., 2018. Toward an ecoregion scale evaluation of eDNA metabarcoding primers: A case study for the freshwater fish biodiversity of the Murray–Darling Basin (Australia). Ecology and Evolution 8, 8697–8712

Faircloth, B.C., Glenn, T.C., 2012. Not all sequence tags are created equal: designing and validating sequence identification tags robust to indels. PLoS One 7(8), e42543.

Guindon, S., Dufayard, J.F., Lefort, V., Anisimova, M., Hordijk, W. and Gascuel, O., 2010. New algorithms and methods to estimate maximum-likelihood phylogenies: assessing the performance of PhyML 3.0. Systematic Biology 59, 307–321.

Hall, T.A., 1999. BioEdit: a user-friendly biological sequence alignment editor and analysis program for Windows 95/98/NT. Nucleic Acids Symposium Series 41, 95–98.

Hammer, M.P., Unmack, P.J., Adams, M., Raadik, T.A., Johnson, J.B., 2014. A multigene molecular assessment of cryptic biodiversity in the iconic freshwater blackfishes (Teleostei: Percichthyidae: *Gadopsis*) of south-eastern Australia. Biological Journal of the Linnean Society 113, 521–540.

Kumar S, Stecher G, Tamura K., 2016. MEGA7: Molecular Evolutionary Genetics Analysis version 7.0 for bigger datasets. Molecular Biology and Evolution 33, 1870–1874.

Lanfear, R., Frandsen, P.B., Wright, A.M., Senfeld, T., Calcott, B., 2016. PartitionFinder 2: new methods for selecting partitioned models of evolution for molecular and morphological phylogenetic analyses. Molecular biology and evolution DOI: dx.doi.org/10.1093/molbev/msw260

Miller M.A., Pfeiffer W., Schwartz T., 2010. Creating the CIPRES Science Gateway for inference of large phylogenetic trees. In: Gateway Computing Environments Workshop, Ieee, 2010. pp 1–8.

Miya, M., Sato, Y., Fukunaga, T., Sado, T., Poulsen, J.Y., Sato, K., Minamoto, T., Yamamoto, S., Yamanaka, H., Araki, H., Kondoh, M., 2015. MiFish, a set of universal PCR primers for metabarcoding environmental DNA from fishes: detection of more than 230 subtropical marine species. Royal Society Open Science 2(7), p.150088.

Richardson, B.J., Baverstock, P.R., Adams, M.A., 1986. Allozyme Electrophoresis. A handbook for animal systematics and population studies. Academic Press, Sydney.

Wickham, H., 2017. Tidyverse: easily install and load 'tidyverse' packages. R package version, 1(1).

Zwickl, D.J., 2006. Genetic algorithm approaches for the phylogenetic analysis of large biological sequence datasets under the maximum likelihood criterion. PhD, The University of Texas.

Table S1. Locality, collection and sample size information for all Hypseleotris specimens used in one more of the molecular studies. Code for each molecular study: PA = primary allozyme dataset; M = mtDNA sequenced; S = SNP dataset; HA = historic allozyme genotyping at key diagnostic loci.

Site	Locality	River Basin	Latitude	Longitude	Field/Freezer code	PA	M	S	HA
1	Drain M, SA	Millicent Coast	-37.393	140.174	FISHLAB-1				12
2	Henry Creek, SA	Millicent Coast	-36.450	139.891	SE01-21				9
3	Back Valley Creek, SA	Lower Murray	-35.541	138.495	FISH_Y4	1	1		15
4	Inman River, SA	Lower Murray	-35.530	138.560	FISHLAB-2	1			9
5	Mundoo Channel, SA	Lower Murray	-35.549	138.915	ML03-61	3			
6	Hindmarsh Island, SA	Lower Murray	-35.528	138.890	FISHLAB-3				4
7	Tookayerta Creek, SA	Lower Murray	-35.429	138.831	FISHLAB-4				22
8	Bull Creek, SA	Lower Murray	-35.295	138.758	FISH84-1				6
9	Dawson Creek, SA	Lower Murray	-35.261	138.871	BERT-1	2	1		338
10	Angas River, Strathalbyn, SA	Lower Murray	-35.258	138.892	FISHLAB-5	1	1		139
11	Angas River, Middle Creek junction, SA	Lower Murray	-35.252	138.891	FISH84-2	1			
12	Bremer River, site #1, SA	Lower Murray	-35.297	139.041	FISHLAB-6		1		12
13	Rodwell Creek, SA	Lower Murray	-35.186	138.911	FISH84-3	2			2
14	Torrens River, SA	Lower Murray	-34.904	138.615	FISHLAB-7	2			25
15	Bremer River, site 2, SA	Lower Murray	-35.008	139.033	IW94-11	2	1		
16	Bremer River, site 3, SA	Lower Murray	-34.980	139.040	FISHLAB-8	1	1		
17	Murray River, Swanport, SA	Lower Murray	-35.153	139.315	PU08-30	1			
18	Murray River, Blanchtown, SA	Lower Murray	-34.349	139.616	PU14-75	4	2		
19	Morgans Lagoon, SA	Lower Murray	-34.038	139.680	FISH_Y2	1	1	1	
20	Murray River, Devlins Pound, SA	Lower Murray	-34.150	140.167	BERT-2/3				42
21	Murray River, Overland Corner, SA	Lower Murray	-34.150	140.330	BERT-4			2	64
22	Loch Luna, SA	Lower Murray	-34.200	140.367	FISHADD6-1				4
23	Murray River, Napper Bridge, SA	Lower Murray	-34.200	140.430	FISHADD6-1				2
24	Eckert Creek, SA	Lower Murray	-34.433	140.533	FISHLAB-9				31

Site	Locality	River Basin	Latitude	Longitude	Field/Freezer code	PA	M	S	HA
25	Yabbie Creek, SA	Lower Murray	-34.430	140.550	BERT-5			2	9
26	Katarapko Creek, SA	Lower Murray	-34.367	140.567	FISHLAB-10				4
27	Salt Creek, SA	Lower Murray	-34.315	140.609	FISHLAB-11		2		85
28	Martins Bend, SA	Lower Murray	-34.283	140.600	FISH99				5
29	Calperum, SA	Lower Murray	-34.200	140.717	FISHLAB-12		2		12
30	Lindsay Island, VIC	Murray	-34.170	141.190	FISHLAB-13	1		1	9
31	Cardross Lakes, VIC	Murray	-34.308	142.095	CARD		1		
32	small creek by Euston cemetary, VIC	Murray	-34.586	142.738	PU14-72	6	2	2	
33	Murray River below Euston Weir, VIC	Murray	-34.603	142.756	PU14-71	2			
34	Black Swamp, Cohuna, VIC	Murray	-35.718	144.189	PU90-00; PU99-34		18		
35	Reedy Lagoon, Cohuna, VIC	Murray	-35.724	144.206	IW94-39/40	4	4		
36	Loddon River, VIC	Loddon	-36.446	143.967	PU13-85	3			
37	Jews Harp Creek, VIC	Campaspe	-37.135	144.586	PU13-74	2			
38	Lake Nagambie, VIC	Goulburn	-36.786	145.134	PU13-70	4			
39	Castle Creek, VIC	Goulburn	-36.784	145.571	PU13-64	6	4	4	
40	Castle Creek, Euroa, VIC	Goulburn	-36.784	145.570	PU15-95			7	
41	Faithfuls Creek, Miepol, VIC	Goulburn	-36.622	145.480	PU15-97			5	
42	Goulburn River, Shepparton, VIC	Goulburn	-36.378	145.397	PU13-62	8	4	4	
43	Ulupna Creek, VIC	Murray	-35.848	145.412	PU08-22	2			
44	Murray River, Bourkes Beach area #1, NSW	Murray	-35.983	145.834	PU14-08	3			
45	Murray River, Bourkes Beach area #2, NSW	Murray	-35.977	145.837	PU17-01			10	
46	Broken River, VIC	Broken	-36.433	145.684	PU13-61	6			
47	creek behind Swanpool, VIC	Broken	-36.756	146.005	PU15-94	2		6	
48	Swanpool Creek, VIC	Broken	-36.746	146.020	PU15-93	2		4	
49	Ovens River, VIC	Ovens	-36.413	146.456	PU13-60	7	1	1	
50	Bight Creek, VIC	Kiewa	-36.373	147.063	PU15-92	2		5	
51	Mitta Mitta River Billabong, VIC	Mitta Mitta	-36.348	147.265	PU09-15/16;PU15-89	6	4	7	
52	Murray River, Leura II Billabong, VIC	Upper Murray	-35.961	147.550	PU13-46B	6			

Site	Locality	River Basin	Latitude	Longitude	Field/Freezer code	PA	M	S	HA
53	Murray River, Billabongs on "Millewa", VIC	Upper Murray	-36.024	147.930	PU13-45B	7	2	2	
54	Murray River, Billabong on "Tinkerry", VIC	Upper Murray	-36.038	147.973	PU13-38B;PU15-87	8		8	
55	Murrumbidgee River, below Balranald Weir, NSW	Murrumbidgee	-34.666	143.491	PU13-40A	2		1	
56	Murrumbidgee River, below Hay Weir, NSW	Murrumbidgee	-34.526	144.712	PU13-38A	5		1	
57	Murrumbidgee River, Wagga Wagga, NSW	Murrumbidgee	-35.105	147.376	PU13-41A	2			
58	Wollundry Lagoon, Wagga Wagga, NSW	Murrumbidgee	-35.110	147.363	PU13-42A	3			
59	Woodbridge Lagoon, Gundagai, NSW	Murrumbidgee	-35.096	148.082	PU13-07	4			
60	Morley Creek, Gundagai, NSW	Murrumbidgee	-35.067	148.107	PU13-06	2			
61	Meadow Creek, Gunning, NSW	Lachlan	-34.779	149.269	PU13-03	18	8	7	
62	Blakney Creek site #1. NSW	Lachlan	-34.586	149.133	PU13-04	5	4	5	
63	Blakney Creek site #2, NSW	Lachlan	-34.646	149.035	PU13-05	1	1	1	
64	Urumwalla Creek site UC05, NSW	Lachlan	-34.670	148.983	UC05		eDNA		
65	Lachlan River, below Cargelligo Weir, NSW	Lachlan	-33.201	146.452	PU16-08			7	
66	Lake Forbes, NSW	Lachlan	-33.350	148.030	PU99-36	4	4	2	
67	Turon River, NSW	Macquarie	-33.073	149.406	PU02-54	2			
68	Dunns Swamp, Rhylstone, NSW	Macquarie	-32.834	150.206	PU99-70	6	6	2	
69	Cudgegong River, NSW	Macquarie	-32.403	149.472	PU14-09	4			
70	Wuuluman Creek, NSW	Macquarie	-32.558	149.075	PU16-74			6	
71	Bogan River, Peak Hill, NSW	Macquarie	-32.723	148.128	PU14-122	2	1	5	
72	Macquarie River, Narromine, NSW	Macquarie	-32.220	148.239	PU14-127	2			
73	Bogan River, Nyngan, NSW	Macquarie	-31.557	147.186	PU90-107;PU14-129	7	2	11	
74	Castlereagh River, Gilgandra, NSW	Castlereagh	-31.714	148.667	PU14-124	4		6	
75	Castlereagh River, Coonabarabran, NSW	Castlereagh	-31.267	149.284	PU14-125			5	
76	Coburn River, Nemingha, NSW	Namoi	-31.125	150.991	PU16-70			4	
77	Namoi River, Manilla, NSW	Namoi	-30.742	150.733	PU14-62	4			
78	Ironbark Creek, ESE of Barraba, NSW	Namoi	-30.400	150.724	PU16-69			4	
79	Cobbedah Creek, N of Cobbedah, NSW	Gwydir	-30.224	150.580	PU16-62			6	
80	Horton River, Upper Horton, NSW	Gwydir	-30.139	150.443	PU16-63			6	

Site	Locality	River Basin	Latitude	Longitude	Field/Freezer code	PA	M	S	HA
81	Macintyre Creek lower, NSW	Gwydir	-30.051	150.780	PU16-59			6	
82	Copeton Dam, NSW	Gwydir	-29.912	150.952	PU14-61	5	2	2	
83	Warialda Creek, Warialda, NSW	Gwydir	-29.540	150.576	PU16-39			4	
84	Gwydir River, near Pallamallawa, NSW	Gwydir	-29.457	150.082	PU13-28	4			
85	Lower Gwydir floodout, Gingham Waterhole, NSW	Gwydir	-29.243	149.305	PU16-38			6	
86	Boomie River, near Neeworra, NSW	Barwon	-29.022	149.064	PU16-37			7	
87	Barwon River, Mungindi, NSW	Barwon	-28.977	148.983	PU14-137	5		8	
88	Barwon River, Collarenebri, NSW	Barwon	-29.547	148.577	PU14-132	5		2	
89	Barwon River, Brewarrina, NSW	Barwon	-29.958	146.857	PU14-134			7	
90	Bogan River, Gongolgon, NSW	Macquarie	-30.347	146.898	PU14-128			1	
91	Darling River, site #1 below Bourke Weir, NSW	Darling	-30.087	145.893	PU14-21	6	2	2	
92	Darling River, site #2 below Bourke Weir, NSW	Darling	-30.086	145.891	PU16-24			4	
93	Willcannia Billabong, NSW	Darling	-31.568	143.398	PU13-17	2		2	
94	Pindari Dam, NSW	Border	-29.388	151.256	PU14-60	1	2		
95	Tenterfield Creek, NSW	Border	-29.030	151.725	PU16-52			10	
96	Deadman Creek, NSW	Border	-29.043	151.822	PU16-53			2	
97	Loghut Creek, NSW	Border	-28.971	151.900	PU16-54			4	
98	Accomadation Creek, QLD	Border	-28.858	151.874	PU14-90	2	1	1	
99	Severn River, Glen Aplin, QLD	Border	-28.740	151.874	PU91-05;PU99-49	5	13		
100	McLaughlin Creek, ESE of Glen Aplin, QLD	Border	-28.775	152.003	PU16-56			4	
101	Dumaresq River, below Bonshaw Weir, QLD	Border	-28.986	151.276	PU14-141			6	
102	Dumaresq River, Texas, QLD	Border	-28.883	151.167	PU97-39	4	1		
103	McIntyre River, Yetman, NSW	Border	-28.900	150.778	PU14-140			12	
104	Condamine River, Killarney, QLD	Condamine	-28.333	152.317	PU97-43	4	2		
105	Condamine River, above Killarney, QLD	Condamine	-28.323	152.342	PU14-12	1			
106	Gap Creek, Main Range, QLD	Condamine	-28.053	152.381	PU91-00		1		
107	Leslie Dam, near Warwick, QLD	Condamine	-28.225	151.921	PU14-14	8	4	4	
108	Jandowae Creek, at Jandowae, QLD	Condamine	-26.779	151.108	PU15-09	4		4	

Site	Locality	River Basin	Latitude	Longitude	Field/Freezer code	PA	M	S	HA
109	Charleys Creek, north of Chinchilla, QLD	Condamine	-26.684	150.801	PU14-40	8	3	3	
110	Condamine River, Chinchilla, QLD	Condamine	-26.800	150.580	PU91-00	1	1		
111	Little Sideling Creek, N of Barakula, QLD	Condamine	-26.245	150.423	PU15-10	4	2	6	
112	Dogwood Creek, Miles, QLD	Condamine	-26.656	150.181	PU15-06			10	
113	Condamine River, at Condamine, QLD	Condamine	-26.926	150.131	PU15-07			5	
114	Bungil Creek, Roma, QLD	Condamine	-26.573	148.800	PU15-05			8	
115	Balonne River, below Beardmore Dam, QLD	Condamine	-27.910	148.649	PU14-138	3			
116	Balonne River, at Lower Plains Station, QLD	Condamine	-28.330	148.387	PU14-139	2		4	
117	Warrego River, Cunnamulla, QLD	Warrego	-28.118	145.687	PU99-63	5	5	5	
118	Langlo River, Bullecourt station, QLD	Warrego	-25.611	145.673	PU15-01			1	
119	Bulloo River, Quilpie, QLD	Bulloo	-26.618	144.278	PU99-62	6	5	6	
120	Barcoo River, Tambo, QLD	Cooper	-24.879	146.257	PU97-103	13	14	5	
121	Barcoo River, Swan Hill, QLD	Cooper	-24.600	145.933	PU91-00	5			
122	Barcoo River, Avington Waterhole, QLD	Cooper	-24.075	144.971	PU15-04			16	
123	Barcoo River, Little Oma Waterhole, QLD	Cooper	-24.277	144.322	PU16-22			2	
124	Aramac Creek, Lynchs Waterhole, QLD	Cooper	-23.125	145.375	PU16-12			5	
125	Lake Dunn, QLD	Cooper	-22.603	145.675	PU14-34	2	2	4	
126	Cooper Creek, Currareva Waterhole, QLD	Cooper	-25.370	142.745	PU14-27	1	1	1	
127	Kyabra Creek, Gummomo Waterhole, QLD	Cooper	-26.097	143.445	PU14-28	2	2	2	
128	Ella Creek, Coongie Lakes, SA	Cooper	-27.017	140.167	ELLA	3	2	2	
129	Brown Creek, Coongie Lakes, SA	Cooper	-27.161	140.164	BROWN	1			
130	Cooper Creek, Narie Waterhole, SA	Cooper	-27.459	140.076	NARI	3	3	2	
131	St Marys Pool, MacDonnell Creek, SA	Cooper	-29.580	139.400	STMAR	5	4	2	
					Totals	306	151	337	860

Table S2. Taxon counts at each site for the three nuclear genetic datasets used on the *Hypseleotris* sexual/unisexual complex. Site numbers follow those used in Figure 2 and Table S1.

	Primary allozyme study						Genomic study						Historic allozyme study					
Site	HA	HAxHB	HAxHX	HB	HBxHX	HX	HA	HAxHB	HAxHX	HB	HBxHX	HX	HA	HAxHB	HAxHX	HB	HBxHX	HX
1													8		4			
2													3		6			
3	1												15					
4	1												9					
5			3															
6													4					
7													5		17			
8														6				
9		2											101	237				
10	1												43	96				
11		1																
12													5	6			1	
13		2												2				
14				2												8	17	
15		1	1															
16					1													
17				1														
18				2	2													
19	1						1											
20													18		10	11	3	
21										2						58	6	
22																3	1	
23																2		
24													22		3	5	1	

	Primary allozyme study						Genomic study						Historic allozyme study					
Site	HA	HAxHB	HAxHX	HB	HBxHX	HX	HA	HAxHB	HAxHX	HB	HBxHX	HX	HA	HAxHB	HAxHX	HB	HBxHX	HX
25										2						2	7	
26															1	3		
27													4		2	63	16	
28													3		2			
29																10	2	
30				1						1						7	2	
32	2		2	2			2											
33					2													
35		2	1		1													
36				1	2													
37				2														
38				2	2													
39	1		4		1		1		2		1							
40							2	3	2									
41							3				2							
42		1	2	3	2			1		2	1							
43					2													
44		1		1	1													
45								2	2	6								
46			1		5													
47		1	1				1	1	4									
48	1				1		2	1	1									
49			1	3	3				1									
50					2				2		3							
51				2	4					5	2							
52				2	4													
53				3	4						2							
54				4	4					4	4							

	Primary allozyme study						Genomic study						Historic allozyme study					
Site	HA	HAxHB	HAxHX	HB	HBxHX	HX	HA	HAxHB	HAxHX	HB	HBxHX	HX	HA	HAxHB	HAxHX	HB	HBxHX	HX
55		1		1			1											
56				2	3						1							
57				2														
58					3													
59				4														
60					2													
61						18						7						
62				2	3					2	3							
63					1						1							
65										4	3							
66					4						2							
67				1	1													
68	4	2					2											
69		1		2	1													
70										4	2							
71	2						3	2										
72				2														
73	2	2	1	1	1			1		4	6							
74				2	2					4	2							
75							2	2			1							
76										4								
77				3	1													
78										4								
79										5	1							
80										4	2							
81										5	1							
82				3	2					2								
83										1	3							

	Primary allozyme study						Genomic study						Historic allozyme study					
Site	HA	HAxHB	HAxHX	HB	HBxHX	HX	HA	HAxHB	HAxHX	HB	HBxHX	HX	HA	HAxHB	HAxHX	HB	HBxHX	HX
84				2	2													
85										2	4							
86										5	2							
87				3	2					5	3							
88				3	2					2								
89										5	2							
90								1										
91				6						2								
92										4								
93				2						2								
94			1															
95								5		2	3							
96								1	1									
97							3	1										
98		2						1										
99	1	1	2		1													
100							2	2										
101										3	3							
102		1		3														
103										8	4							
104					4													
105	1																	
107	3	4		1			2	1		1								
108	2	2					1	3										
109	2	2			4		2	1										
110					1													
111	4						5	1										
112							2	5	3									

	Primary allozyme study						Genomic study						Historic allozyme study					
Site	HA	HAxHB	HAxHX	HB	HBxHX	HX	HA	HAxHB	HAxHX	HB	HBxHX	HX	HA	HAxHB	HAxHX	HB	HBxHX	HX
113										3	2							
114							2	2		3	1							
115				2	1													
116				2						4								
117				2	3					2	3							
118										1								
119				3	3					4	2							
120				10	3					3	2							
121				5														
122										7	9							
123										2								
124										5								
125				2						4								
126				1						1								
127				2						2								
128				1	2						2							
129				1														
130				3						2								
131				5						2								
Totals	29	29	20	115	95	18	39	37	18	151	85	7	240	347	45	172	56	0

Table S3. Allozyme profiles for all *Hypseleotris* sexual and unisexual taxa. Profiles in black font are shown as allele frequencies (as a superscript percentage), while those in red font indicate the observed numbers of each bi-allelic genotype in hybridogens for the loci which are fixed or nearly-fixed between the two parental taxa. Invariant loci: *Acp*, *Ald1*, *Ald2*, *Gp1*, *Gpd*, *Ldh1*, and *Ldh2*.

**Locus**	**HA (29)**	**HB (MDB) (82)**	**HB (inland) (33)**	**HX (18)**	**HA x HB* (29)**	**HA x HX* (20)**	**HB x HX* (MDB) (87)**	**HB x HX* (inland) (8)**
Acon	d^{96},c^{2},a	e^{70},g^{27},d^{2},f	e	e^{69},d	$de^{19}/dd^{5}/be^{3}$	$de^{16}/ce,ee,ae^{1}$	e^{87},g^{12},d	e
Ada	e^{62},c^{36},b	g	g	f	$eg^{22}/cg^{5}/ee^{2}$	$ef^{16}/cf^{2}/af,df^{1}$	fg^{87}	fg^{8}
Adh1	b	b	b	b	b^{98},a	b^{97},a	b^{95},c	b
Adh2	d	d^{88},b	d	d	d^{93},b	$d^{88},a^{5},b^{3},c^{2},e$	d^{91},b	d
Ak	a^{98},b	a	a	a	a	a	a	a
Aldh	b^{97},c	b	b	b	b	b^{88},d^{10},c	b^{96},a^{2},d	b^{81},a
Ca	$c^{57},d^{29},a^{5},f^{5},e^{2},b$	f	f	c	$cf^{14}/df^{7}/ff^{5}/bf^{1}$	c^{92},b^{3},d^{3},f	cf^{86}/ff^{1}	cf^{8}
Ck	b	b	b	b	b^{97},a	b	b	b
Eno1	e^{95},c	$e^{88},c^{7},f^{2},a^{1},h^{1},i$	e^{85},i^{8},j	e	e^{93},f^{5},c	e^{90},c^{5},a^{3},d	$e^{83},c^{9},f^{6},b^{1},a$	e^{81},i^{7},c^{6},j
Eno2	a	a	a	a	a	a^{98},b	a	a
Est1	c^{74},a^{22},b^{2},d	d	d^{98},c	d^{75},c	$cd^{18}/ad^{10}/cc^{1}$	c^{65},d^{23},f^{7},a	d^{75},c	d^{69},c
Est2	a^{98},b	a	a	a	a	a	a	a
Fdp	b^{95},a	b	b^{98},a	c	b^{97},a	bc^{20}	bc^{87}	bc^{7}/bb^{1}
Fum	a	b^{99},c	b	b	ab^{28}/bb^{1}	ab^{19}/bb^{1}	b	b
G6pd	a	b^{99},a	b	b	ab^{29}	ab^{19}	b	b
Gapd	a	a	a	a	a	a^{98},b	a	a
Glo	c^{98},a	d^{78},e^{16},b^{5},c	e	c	$cd^{24}/ce^{3}/bc,cc^{1}$	c	$cd^{69}/ce^{11}/bc^{5}/cc^{2}$	ce^{8}
Got1	b	b	b	a	b	ab^{20}	ab^{87}	ab^{8}
Got2	d^{88},b^{5},g^{5},f	d^{99},g	e^{98},d	d	d^{96},h	d^{97},c	d^{93},b^{5},c^{1},a	de^{8}
Gp2	b^{91},a	b	b	a	b^{98},a	ab^{19}/aa^{1}	ab^{87}	ab^{8}
Gp3	b^{98},a	a	a	a	ab^{22}/aa^{6}	ab^{17}/aa^{3}	a	a
Gpi1	c^{66},e^{31},f	e^{74},c^{18},a	a	c	e^{45},c^{44},a^{9},b	c^{98},e	c^{60},e^{36},a^{3},d	ac^{8}
Gpi2	b^{97},a^{2},c	d	b	d	bd^{29}	bd^{19}/ad^{1}	d^{99},e	bd^{5}/bb^{3}
Gsr	d^{88},c	a^{99},b	a	d	$ad^{27}/cd,dd^{1}$	d^{97},c	ad^{87}	ad^{8}

Locus	HA (29)	HB (MDB) (82)	HB (inland) (33)	HX (18)	HA x HB* (29)	HA x HX* (20)	HB x HX* (MDB) (87)	HB x HX* (inland) (8)
Idh1	b^{98},a	b^{99},a	b	b	b	b	b	b
Idh2	b^{98},c	b	a	b	b	b	b	ab^{8}
Lap	b	a^{46},b^{44},c^{10}	b	b	b^{74},a^{26}	b^{92},a^{8}	b^{69},a^{26},c^{5}	b
Mdh1	b^{93},a	b	b	b	b^{98},a	b	b	b
Mdh2	a	a^{99},b	a	a	a	a^{98},b	a	a
Mdh3	a	a	a	a	a	a	a	a^{75},b
Me	e^{78},g	e^{99},c	e	d^{78},b	e^{93},g	$de^{12}/dg^{7}/ee^{1}$	de^{86}/be^{1}	de,ae^{4}
Mpi	c^{39},d^{24},a^{21},g^{12},e^{2},b	h^{99},f	h	h	$ch^{17}/dh^{6}/ah^{5}/bh^{1}$	$ch^{12}/dh^{7}/ah^{1}$	h^{99},i	h
Ndpk	a	a^{99},b	a	a	a	a	a	a
PepA	a^{97},b	a^{99},b	a	c	a^{98},b	ac^{19}/aa^{1}	$ac^{83}/ad^{3}/cc^{1}$	ac^{8}
PepB	g^{86},d^{10},b^{2},h	d^{58},a^{32},c^{9},b	c^{92},d	d^{53},c	$cg^{11}/ag^{6}/dg^{5}/bg^{4}/cd^{2}/ad^{1}$	d^{50},g^{45},e^{3},h	d^{73},a^{17},c^{9},f	c^{50},d
PepC	b^{88},a^{10},d	a^{59},b	a^{98},b	d	b^{95},a^{3},c	bd^{19}/be^{1}	$ad^{38}/bd^{26}/bb^{10}/aa^{9}/ab^{4}$	ad^{7}/ab^{1}
PepD1	e	d^{99},c	d	b	de^{29}	be^{19}/bb^{1}	$bd^{74}/dd^{12}/cd^{1}$	bd^{5}/ad^{3}
PepD2	b	c^{85},d	c	c	$bc^{24}/ac^{3}/bd^{2}$	bc^{20}	c^{94},d	c
Pgam	b^{95},a	b^{96},a	b	b	b^{98},a	b	b^{85},a	b
6Pgd	b^{98},a	b	b	b	b	b^{90},a^{8},d	b^{99},c	b
Pgk	c	c^{99},d	c	c	c^{91},a	c^{97},a	c^{98},b^{1},a	c
Pgm	b^{60},c^{33},a	c^{88},b	b	c	c^{53},b^{45},a	c^{77},b	c^{77},d^{15},b	bc^{8}
Pk1	b	b	b^{97},a	b	b	b	b	b
Pk2	b	b^{99},d	b	b	b	b	b^{99},c	b^{94},a
Sordh	b^{81},a	a	a	a	a^{64},b	a^{53},b	a	a
Tpi1	a	a	a^{98},b	a	a	a	a	a
Tpi2	c^{72},b	c^{77},b	b^{74},c	c	c^{77},b^{21},a	c^{95},b	c^{86},b	b^{50},c
H_O	0.071	0.069	0.015	0.032	0.320	0.354	0.280	0.365
±S.E.	±0.015	±0.019	±0.007	±0.016	±0.056	±0.059	±0.053	±0.061

Table S4. Summary of pairwise genetic differences among taxa for the allozyme dataset. Lower matrix = number of fixed differences (allowing a cumulative tolerance for shared alleles of up to 10%). Upper matrix = unbiased Nei's Distance. Values involving hybridogenetic lineages and their parental ancestors or sexually-parasitised congener are shown in red, and further demonstrate their genetic intermediacy.

Taxon	HA (39)	HB MDB (119)	HB inland (32)	HX (7)	HAxHB* (37)	HAxHX* (18)	HBxHX* MDB (70)	HBxHX* inland (15)
HA	-	0.32	0.37	0.37	0.07	0.09	0.29	0.27
HB (MDB)	13	-	0.13	0.26	0.08	0.21	0.06	0.10
HB (inland)	16	4	-	0.38	0.17	0.32	0.19	0.07
HX	15	11	16	-	0.24	0.08	0.07	0.10
HAxHB*	0	0	5	8	-	0.08	0.10	0.11
HAxHX*	0	5	10	0	0	-	0.08	0.10
HBxHX* MDB	10	0	6	0	0	0	-	0.04
HBxHX* inland	9	0	0	0	1	0	0	-
